# Association between family income to poverty ratio and HPV infection status among U.S. women aged 20 years and older: a study from NHANES 2003-2016

**DOI:** 10.3389/fonc.2023.1265356

**Published:** 2023-10-04

**Authors:** Yuan Zhao, Jing Zhao, Ruijie Xie, Yu Zhang, Ya Xu, Jing Mao, Cheng Yan, Yi Sun

**Affiliations:** ^1^ Department of Clinical Laboratory, The First People's Hospital of Yunnan Province, The Affiliated Hospital of Kunming University of Science and Technology, Kunming, China; ^2^ Department of Gynecology, Hebei General Hospital, Shijiazhuang, China; ^3^ The Affiliated Nanhua Hospital, Hengyang Medical School, University of South China, Hengyang, China; ^4^ Department of Clinical Laboratory, The First People’s Hospital of Yunnan Province, Kunming, China

**Keywords:** HPV, NHANES, family income to poverty ratio, association, US

## Abstract

**Background:**

HPV infection is closely related to the occurrence of cervical cancer and has an important adverse effect on human life and health. This study used data from the NHANES 2003–2016 to investigate the relationship between PIR and HPV infection status among Americans aged 20 and older.

**Methods:**

The data for this cross-sectional investigation came from the 2003–2016 National Health and Nutrition Examination Survey (NHANES), which included 9580 women who were 20 years of age or older. The linear and nonlinear correlations between PIR and the presence of HPV infection were investigated using multiple linear regression and smooth curve fitting. The stability of the relationship across groups was examined using subgroup analysis and interaction tests.

**Results:**

There were 2232 impoverished homes and 2543 rich households among the 9580 adult participants aged 20 and above. PIR (ratio of income to poverty) was found to be significantly inversely related to the presence of HPV infection [0.91 (0.89, 0.94)] after adjusting for all other covariates, and the trend persisted even after categorizing PIR into high- and low-income groups (PIR>4 and PIR<1). In addition, significant negative relationships were discovered in subgroup analyses for women aged 25 to 59 [0.90 (0.88, 0.93)], non-Hispanic whites [0.80 (0.70, 0.92)], non-diabetics [0.91 (0.88, 0.94)], and those who had ever engaged in sex [0.91 (0.89, 0.94)].

**Conclusions:**

PIR was highly and negatively correlated with the presence of HPV infection in American women aged 20 and older. The results of this study are of great significance for preventing HPV infection and improving the accuracy of HPV screening.

## 1 Introduction

Persistent infection with the human papillomavirus (HPV) can lead to cervical cancer, which kills women worldwide, and is the most common sexually transmitted infection (STI) (1, 2). The prevalence of HPV infection has been connected to several illnesses, including oropharyngeal and anogenital malignancies, and among oropharyngeal cancers, which some studies claim to have the fastest incidence rate among all cancers in high-income countries, while the latest study demonstrated that low-income was correlated with HPV infection and cervical cancer (3, 4). Therefore, research on the correlation between income and HPV infection is very necessary.

Based on statistics, there are 80 million HPV-positive individuals in the United States, and HPV oropharyngeal cancer is more prevalent than HPV cervical cancer (5). According to one report, young women between the ages of 20 and 24 had the highest incidence of HPV infection in the U.S (6). More than 80 different forms of HPV have been identified, of which the most dangerous variants 16 and 18 are responsible for 70% of cases of cervical cancer. Additionally, E5, E6, and E7 are where the majority of HPV’s pathogenicity is expressed, which can cause low- to high-grade cervical lesions (CIN-1, 2, 3) (7). The pathogenesis of cervical cancer due to HPV infection is now well-established, and there are four main stages in the growth of cervical cancer: invasion through the epithelial cell basement membrane, viral persistence, progression of the continuously infected epithelium to the cervical precursor, and infection of the pyogenic epithelium in the region of transformation of the cervix (8). Finding signs that can indicate if you have an HPV infection is therefore especially important.

Family income to poverty ratio (PIR) is closely related to the health of human life, and it has been claimed that PIR can affect children’s health, especially among low-income children in the U.S., confirming that higher family incomes are significantly correlated with lower child morbidity rates (9). In addition, PIR has been significantly correlated with abdominal obesity in U.S. women (10), substantial and adversely linked with the severity of hepatic steatosis in teenagers in the United States (11), and it has been demonstrated that youngsters in low- and middle-income households in the neighborhood may be more susceptible to sadness and anxiety (12). Furthermore, compared to children from wealthy homes, children from families with low revenues had considerably worse lung function (13). Previous studies have confirmed that PIR affects every aspect of our lives. The causes of HPV are complex, and there may be an interplay of many factors, such as social, human, and genetic factors. Calcium intake may negatively associate with HPV infection status (14). The transmission of HPV and cardiovascular disease are linked. In women who had received an HPV vaccine, the link was insignificant (15). Lower household incomes have been found in previous studies to lead to poorer health protection, including a lack of access to timely and effective disease prevention and diagnosis, which can lead to the development of many diseases (16), including cancer (17). Most poor people are unable to get timely HPV screening and HPV vaccination for economic reasons, which is one of the major reasons for HPV in low-income households (18). One study claimed that low-income families had higher incidence and mortality rates for cancer, which included cervical cancer, but the study was limited to the Florida area (19). Although there have been several prior research on HPV, no more in-depth studies have explored the relationship between PIR and HPV infection status.

In order to investigate the relationship between family income to poverty ratio and HPV infection status among American women aged 20 and older, we conducted cross-sectional research utilizing data from the NHANES 2003–2016.

## 2 Methods

### 2.1 Study populations

**Figure 1:**
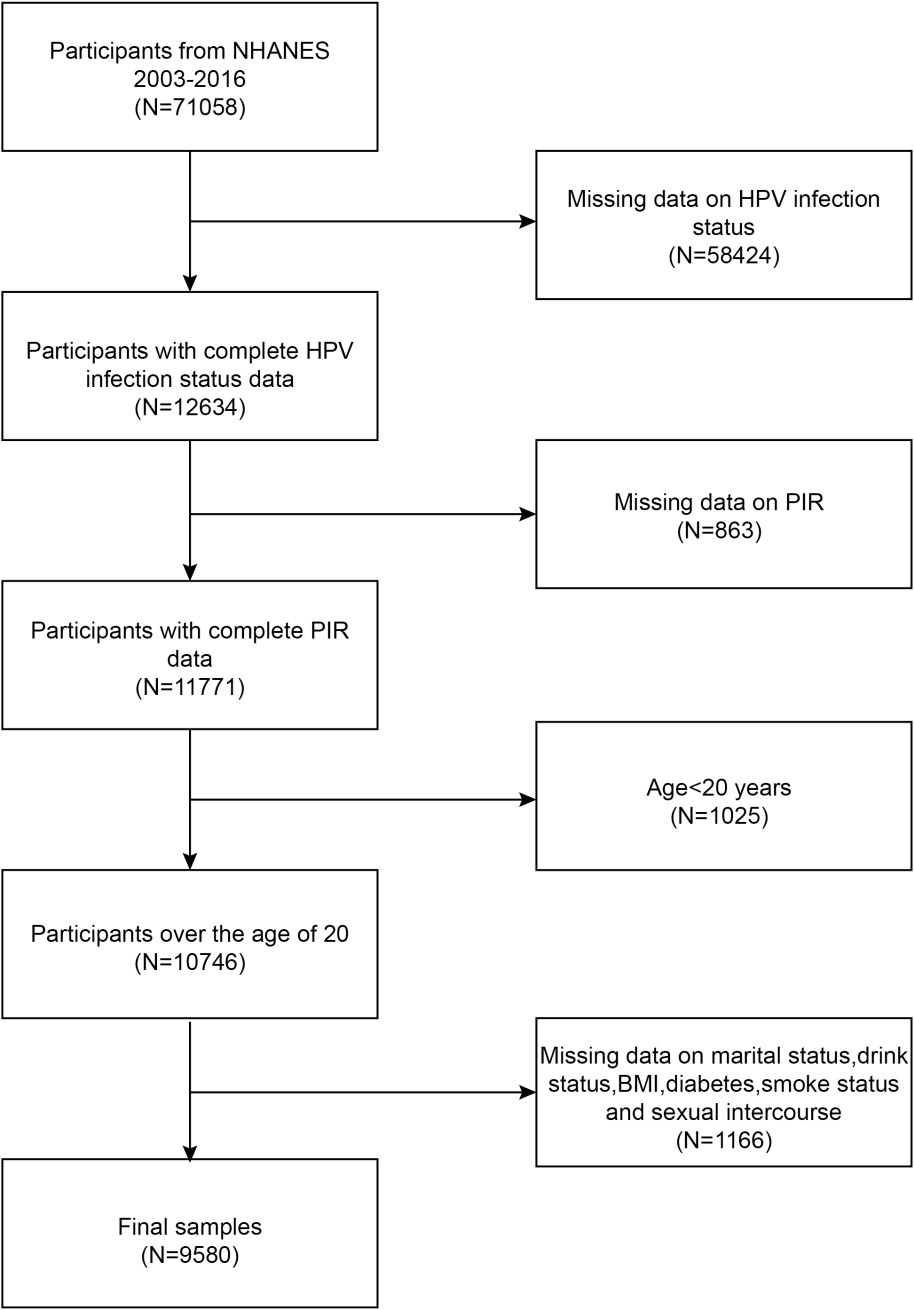
Flowchart of participant selection. NHANES, National Health and Nutrition Examination Survey; PIR, ratio of family income to poverty; BMI, body mass index; HPV, human papillomavirus.

The National Health and Nutrition Examination Study (NHANES) is a population-based, cross-sectional study used to gather data on the health and nutrition of Americans living in households. The study procedure was approved by the National Center for Health Statistics (NCHS) Research Ethics Review Board. All subjects gave their written consent at the time of recruiting. Statistics for this research came from the NHANES during 2003-2016. In this survey, we excluded 58424 participants with missing HPV infection status, 863 participants with missing PIR, 1025 participants under the age of 20 years, and 1166 participants with missing status for the remaining covariates. The study ultimately included 9580 participants. The workflow for choosing samples is shown in **Figure 1**.

### 2.2 Study variables

The PIR was the study’s exposure variable, while the outcome variable was the presence of HPV infection. We track the Consumer Price Index (CPI) based on family incomes and federally recognized levels of poverty to determine the relevant income index, PIR, which we divide into three classes: the poor (PIR<1), the middle class (PIR1-4), and the rich (PIR>4). HPV infection status was determined by genotyping DNA extracted from vaginal swabs, obtained by the Digene hybridization capture method and prototype line blot assay and Roche Linear Array.

Age, race, marital status, smoking, consumption of alcohol, sexual intercourse, diabetic status, and BMI were all covariates in this study. On the NHANES website’s official page (https://www.cdc.gov/nchs/nhanes), you may find explanations for all variables.

### 2.3 Statistical analysis

R (version 4.1.3) and Empower Stats (version 2.0) were used for all analyses. Demographic characteristics of subjects were assessed by PIR categorization using a t-test and chi-square test. The linear association between PIR and HPV infection status was examined using multiple regression models. The nonlinear association between PIR and HPV infection status was examined using the weighted smoothed curve fitting approach and the generalized summation model. By using subgroup analysis and an interaction test, the connection between PIR and HPV infection status in various categories was investigated.

## 3 Results

### 3.1 Baseline characteristics

By PIR, the weighted features were divided into three groups: low-income, medium-income, and high-income. There were 9580 adult females above the age of 20, and the mean PIR for everyone who took part was 2.48 ± 1.67. The high-income group’s participants (PIR > 4) were older than the low-income group’s (PIR<1) participants. Adult females living below the poverty line had a higher likelihood of being Mexican American and non-Hispanic black and having higher BMI levels than adult females living in affluent households. (**Table 1**).

**Table 1:** Basic characteristics of participants.

Variables	Low income (PIR<1,N=2232)	Middle income (PIR 1-4,N=4805)	High income (PIR≥4,,N=2543)	*P*-value
Age (years)	36.25 ± 11.66	38.56 ± 11.30	41.59 ± 10.84	<0.0001
Race (%)				<0.0001
Mexican American	25.36	18.76	7.57	
Other Hispanic	10.24	10.25	5.64	
Non-Hispanic White	33.40	40.10	58.13	
Non-Hispanic Black	24.82	22.85	16.18	
Other Race	6.18	8.03	12.48	
Marital status (%)				<0.0001
Married	29.41	48.98	67.97	
Widowed	2.81	2.36	0.62	
Divorced	14.53	12.08	8.78	
Separated	7.19	4.38	1.19	
Never married	32.07	22.19	15.19	
Living with partner	13.99	10.00	6.24	
Drink status(%)				<0.0001
Yes	60.44	64.92	76.30	
No	39.56	35.08	23.70	
Smoke status(%)				<0.0001
Yes	44.15	36.83	31.94	
No	55.85	63.17	68.06	
Sexual intercourse(%)				<0.0001
Yes	95.08	96.83	97.36	
No	4.92	3.17	2.64	
Diabetes(%)				<0.0001
Yes	9.02	6.97	4.20	
No	89.66	91.40	94.43	
Borderline	1.32	1.63	1.37	
BMI (kg/m^2^)	30.79 ± 8.51	29.76 ± 7.81	27.85 ± 7.11	<0.0001
HPV infection status (%)				<0.0001
Positive	53.39	44.74	34.31	
Negative	46.61	55.26	65.69	

Mean±SD for continuous variables: the P value was calculated by weighted linear regression model.

(%)for categorical variables:the P value was calculated by the weighted chi-square test.

PIR, the ratio of family income to poverty; BMI, body mass index; HPV, human papillomavirus.

### 3.2 Relationship between PIR and HPV status

**Figure 2:**
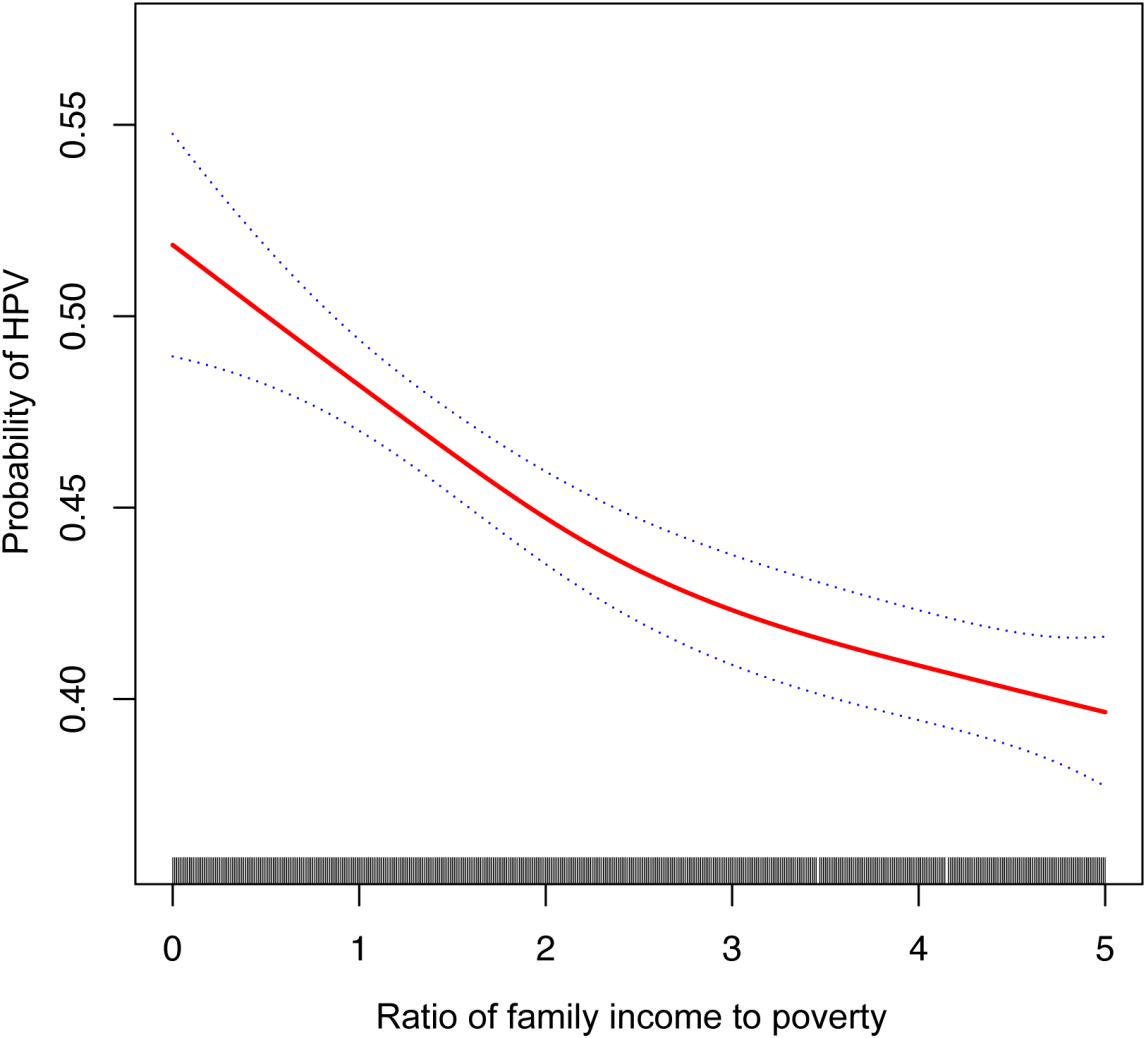
The solid red line represent the smooth curve fit between variables. Blue bands represent the 95% of confidence interval from the fit. Controles Attenuation Parameter; Ratio of family income to poverty, PIR.

The findings of multivariate linear regression analysis for all three models of the association between PIR and HPV infection status are presented in **Table 2**. The three models (model I, OR= 0.83, 95% CI: 0.81, 0.86; model II, OR= 0.91, 95% CI: 0.89, 0.94; and model III, OR= 0.91, 95% CI: 0.89, 0.94) all demonstrated a significant negative connection between PIR and HPV infection status. There was a significant difference in the trend of the high-income group (PIR>4) compared to the low-income group (PIR1), which served as the control group, after dichotomizing the PIR of the variables. In addition, we tried to fit a smoothed curve to the nonlinear connection between PIR and HPV infection status, and the outcomes showed that there is a nonlinear negative association between the two variables (**Figure 2**).

**Table 2:** The associations between family PIR and HPV infection status.

Exposure	Model I OR (95% CI) *P*	Model II OR (95% CI) *P*	Model III OR (95% CI) *P*
Ratio of family income to poverty	0.83 (0.81,0.86) <0.0001	0.91 (0.89, 0.94) <0.0001	0.91 (0.89, 0.94) <0.0001
PIR classification			
Low income(PIR<1)	Reference	Reference	Reference
Middle income(PIR 1-4)	0.70 (0.63, 0.77) <0.0001	0.83 (0.74, 0.92) 0.0005	0.82 (0.74, 0.92) 0.0004
High income(PIR≥4)	0.46 (0.41, 0.52) <0.0001	0.70 (0.61, 0.79) <0.0001	0.69 (0.61, 0.79) <0.0001
*P* for trend	<0.0001	<0.0001	<0.0001

Model I: None covariates were adjusted;

Model II: Age, race, and marital status were adjusted;

Model III: Age, race, marital status, drink status, smoke status, sexual intercourse, diabetes, and BMI were adjusted.

PIR, the ratio of family income to poverty; BMI, body mass index; HPV, human papillomavirus.

### 3.3 Subgroup analyses

We discovered erratic correlations between PIR and HPV infection status in subgroup analyses stratified by age, race, diabetes, and sexual activity. As shown in **Table 3**, although the relationship between PIR and HPV infection status was negatively correlated in the vast majority of subgroups, this linear negative correlation was only significant among female participants aged 25-29 years, other Hispanic,non-Hispanic whites, non-Hispanic blacks, nondiabetics, and participants who had had sexual intercourse. More crucially, the findings of the interaction test revealed no evidence of a connection between this unfavorable correlation and age, race, diabetes, or sexual activity.

**Table 3:** Subgroup analysis of the association between family PIR and HPV infection status.

Subgroup	OR(95%CI)	*P* for interaction
Age		0.628
20-24	0.92 (0.85, 1.00)	
25-59	0.90 (0.88, 0.93)	
Race		0.147
Mexican American	1.01 (0.93, 1.09)	
Other Hispanic	0.90 (0.81, 0.99)	
Non-Hispanic White	0.90 (0.86, 0.94)	
Non-Hispanic Black	0.91 (0.86, 0.97)	
Other Race	0.90 (0.82, 1.00)	
Diabetes status		0.172
Yes	1.01 (0.90, 1.14)	
No	0.91 (0.88, 0.94)	
Borderline	0.85 (0.65, 1.11)	
Sexual intercourse		0.819
Yes	0.91 (0.89, 0.94)	
No	0.89 (0.73, 1.09)	

Age, race, marital status, drink status, smoking status, sexual intercourse, diabetes, and BMI were adjusted. In the subgroup analyses, the model is not adjusted for the stratification variable itself.

PIR, the ratio of family income to poverty; BMI, body mass index; HPV, human papillomavirus.

## 4 Discussion

In this research, we sought to define the relationship between PIR and HPV infection status in American women 20 years of age and older. Upon examination, we discovered a strong inverse relationship between PIR and the presence of HPV infection [0.91 (0.89, 0.94)], and smoothed curve fitting revealed a non-linear negative association between the two. Age, race, diabetes, and sexual activity did not significantly depend on the adverse connection, according to subgroup analyses and interaction results.

Previous research has shown that HPV is linked to several ailments, and a recent study called Estimating the Global Prevalence of Human Papillomavirus Infection in Pregnant Women found that young age with low levels of education, multiple lifelong partners, and young age with low levels of HIV infection were all associated with increased risk. Pregnant women also had a significantly higher prevalence of pregnancy-related illnesses and HIV than the general population (20). Moreover, studies have also demonstrated that smoking, HIV and herpes infections are also significant risk factors for cervical cancer, which is consistent with our study (21). However, we are also well aware that HPV infection is connected to a variety of factors, such as social, human, and genetic factors. Among these many factors, the household income poverty ratio (HIPR) comes to mind first, as it has been shown in many studies that PIR is inextricably linked to a wide range of diseases, that the lower the household income, the greater the negative impact on children’s health (22), and in particular the higher the risk of cancer (23), and that housing affordability is significantly correlated with the incidence of severe maternal morbidity (24). Inequalities in socio-economic status may lead to different likelihoods of disease occurrence, such that groups with higher household incomes can respond faster and more efficiently at an earlier stage of disease recognition, while groups with lower household incomes are more at risk due to a lack of awareness (25, 26).

Furthermore, high-income nations adopt cytology-based screening techniques, which produce more accurate screening findings (27, 28). Screening and HPV vaccine are crucial preventative measures against HPV infection, in addition to avoiding multiple sexual partners (29, 30). However, this is difficult for low-income households. Consistently high rates of HPV infection—which are most pronounced, especially in developing nations—are caused by a lack of adequate economic levels, a lack of understanding of public health levels, and a lack of access to the necessary anti-cancer medicines (31). But more research has to be done to determine the processes behind the link between PIR and HPV infection status.

There are still shortcomings in our study, first of all, as it is a cross-sectional analysis, we are unable to discuss the causal link and the underlying mechanisms of effect, and secondly, the covariates included may not be completely accurate which can affect the accuracy of the results. However, there are some strengths in our study, such as the fact that It is founded on a great deal of representative data from NHANES, and the fact that we have demonstrated a non-linear negative association between PIR and HPV status, which has diagnostic value in predicting the presence of HPV in a patient.

PIR was strongly and unfavorably related to the presence of HPV infection in American women aged 20 and older. Population variations in PIR should be taken into consideration during HPV diagnosis and therapy. PIR is anticipated to play a significant role in the population’s propensity to HPV infection. Improving the income level of patients and strengthening the screening of HPV in poverty-stricken areas may contribute to the prevention and treatment of cervical cancer. Future research is required to corroborate our findings.

## Data availability statement

The datasets presented in this study can be found in online repositories. The names of the repository/repositories and accession number(s) can be found in the article/supplementary material.

## Ethics statement

The studies involving humans were approved by NCHS Ethics Review Board (ERB) Approval. The studies were conducted in accordance with the local legislation and institutional requirements. Written informed consent for participation in this study was provided by the participants’ legal guardians/next of kin.

## Author contributions

YuaZ: Writing – original draft, Conceptualization, Data curation, Investigation, Methodology, Software, Writing – review & editing. JZ: Investigation, Writing – review & editing. RX: Data curation, Writing – review & editing. YuZ: Methodology, Writing – review & editing. YX: Conceptualization, Writing – review & editing. JM: Investigation, Writing – review & editing. CY: Investigation, Writing – review & editing. YS: Funding acquisition, Resources, Supervision, Writing – review & editing.
